# Supplementary material for: MetaRibo-Seq measures translation in microbiomes
Source: Nat Commun. 2020 Jun 29;11:3268. doi: 10.1038/s41467-020-17081-z (PMC7324362; doi:10.1038/s41467-020-17081-z)
Supplement: Supplementary file 10 — Supplementary Data 7 [file 41467_2020_17081_MOESM10_ESM.zip › File2/Confidence_High_Taxonomy/394901_out.krona.html]

Javascript must be enabled to view this page.

members
magnitude
magnitudeUnassigned
count
unassigned
taxon
rank

394901\_out

96

96
superkingdom
2

phylum
976
96

200643
class
96

96
171549
order

815
family
96

96
1

SRS021484\_contig\_number\_3806
816
genus


SRS144506\_contig\_number\_42179
1
species
371601

species
821

SRS011084\_contig\_number\_29337
1

species
817

SRS012902\_contig\_number\_989SRS013158\_contig\_number\_1374SRS013215\_contig\_number\_contig-100\_554.18188SRS014459\_contig\_number\_4190SRS014613\_contig\_number\_12887SRS014736\_contig\_number\_2581SRS015065\_contig\_number\_35838SRS015190\_contig\_number\_14386SRS015264\_contig\_number\_10836SRS015578\_contig\_number\_25561SRS015890\_contig\_number\_12424SRS015960\_contig\_number\_12793SRS016132\_contig\_number\_4279SRS016267\_contig\_number\_8110SRS016495\_contig\_number\_8913SRS017433\_contig\_number\_1587SRS017521\_contig\_number\_33866SRS017622\_contig\_number\_636SRS018313\_contig\_number\_7167SRS018541\_contig\_number\_8854SRS018817\_contig\_number\_9725SRS018888\_contig\_number\_4879SRS018984\_contig\_number\_16919SRS019267\_contig\_number\_5960SRS019397\_contig\_number\_1665SRS019601\_contig\_number\_11535SRS019787\_contig\_number\_8182SRS022524\_contig\_number\_6025SRS023346\_contig\_number\_3430SRS023829\_contig\_number\_145SRS023971\_contig\_number\_4392SRS024132\_contig\_number\_13952SRS024388\_contig\_number\_10747SRS024549\_contig\_number\_1174SRS043411\_contig\_number\_12436SRS043701\_contig\_number\_16228SRS046502\_contig\_number\_10030SRS046712\_contig\_number\_270SRS048981\_contig\_number\_1277SRS049402\_contig\_number\_3547SRS050299\_contig\_number\_9151SRS051610\_contig\_number\_7585SRS053214\_contig\_number\_5829SRS053573\_contig\_number\_104SRS055966\_contig\_number\_1474SRS057717\_contig\_number\_1649SRS058723\_contig\_number\_6005SRS063040\_contig\_number\_14895SRS063127\_contig\_number\_contig-100\_63.32111SRS063190\_contig\_number\_9379SRS063985\_contig\_number\_15146SRS064276\_contig\_number\_15239SRS074964\_contig\_number\_6102SRS075021\_contig\_number\_967SRS075398\_contig\_number\_1021SRS076804\_contig\_number\_17898SRS077194\_contig\_number\_7211SRS077335\_contig\_number\_4066SRS077589\_contig\_number\_2871SRS077730\_contig\_number\_11026SRS078419\_contig\_number\_14103SRS097920\_contig\_number\_11241SRS098655\_contig\_number\_748SRS098717\_contig\_number\_18543SRS098881\_contig\_number\_13725SRS104036\_contig\_number\_7210SRS1041039\_contig\_number\_15208SRS1041145\_contig\_number\_15955SRS104311\_contig\_number\_20604SRS104485\_contig\_number\_803SRS104636\_contig\_number\_18238SRS1054928\_contig\_number\_6210SRS1055043\_contig\_number\_385SRS1055069\_contig\_number\_13585SRS1055099\_contig\_number\_5560SRS140492\_contig\_number\_10377SRS140513\_contig\_number\_9288SRS142599\_contig\_number\_25967SRS143342\_contig\_number\_11890SRS143417\_contig\_number\_19795SRS144135\_contig\_number\_28098SRS144362\_contig\_number\_2814SRS146812\_contig\_number\_49787SRS147022\_contig\_number\_1648SRS147139\_contig\_number\_33213SRS147272\_contig\_number\_206SRS147377\_contig\_number\_5166SRS147653\_contig\_number\_4136SRS148159\_contig\_number\_27106SRS893279\_contig\_number\_1912SRS893342\_contig\_number\_2098SRS971275\_contig\_number\_23712
92

species
46506

SRS056259\_contig\_number\_4245
1
